# A QTL for Number of Teats Shows Breed Specific Effects on Number of Vertebrae in Pigs: Bridging the Gap Between Molecular and Quantitative Genetics

**DOI:** 10.3389/fgene.2019.00272

**Published:** 2019-03-26

**Authors:** Maren van Son, Marcos S. Lopes, Henry J. Martell, Martijn F. L. Derks, Lars Erik Gangsei, Jorgen Kongsro, Mark N. Wass, Eli H. Grindflek, Barbara Harlizius

**Affiliations:** ^1^Norsvin SA, Hamar, Norway; ^2^Topigs Norsvin Research Center, Beuningen, Netherlands; ^3^Topigs Norsvin, Curitiba, Brazil; ^4^School of Biosciences, University of Kent, Canterbury, United Kingdom; ^5^Department of Animal Sciences, Wageningen University and Research, Wageningen, Netherlands; ^6^Animalia AS, Oslo, Norway; ^7^Faculty of Chemistry, Biotechnology and Food Sciences, Norwegian University of Life Sciences, Ås, Norway

**Keywords:** teats, vertebrae, ribs, QTL, fine mapping, spine development, mammary gland, SSC7

## Abstract

Modern breeding schemes for livestock species accumulate a large amount of genotype and phenotype data which can be used for genome-wide association studies (GWAS). Many chromosomal regions harboring effects on quantitative traits have been reported from these studies, but the underlying causative mutations remain mostly undetected. In this study, we combine large genotype and phenotype data available from a commercial pig breeding scheme for three different breeds (Duroc, Landrace, and Large White) to pinpoint functional variation for a region on porcine chromosome 7 affecting number of teats (NTE). Our results show that refining trait definition by counting number of vertebrae (NVE) and ribs (RIB) helps to reduce noise from other genetic variation and increases heritability from 0.28 up to 0.62 NVE and 0.78 RIB in Duroc. However, in Landrace, the effect of the same QTL on NTE mainly affects NVE and not RIB, which is reflected in reduced heritability for RIB (0.24) compared to NVE (0.59). Further, differences in allele frequencies and accuracy of rib counting influence genetic parameters. Correction for the top SNP does not detect any other QTL effect on NTE, NVE, or RIB in Landrace or Duroc. At the molecular level, haplotypes derived from 660K SNP data detects a core haplotype of seven SNPs in Duroc. Sequence analysis of 16 Duroc animals shows that two functional mutations of the Vertnin (*VRTN*) gene known to increase number of thoracic vertebrae (ribs) reside on this haplotype. In Landrace, the linkage disequilibrium (LD) extends over a region of more than 3 Mb also containing both *VRTN* mutations. Here, other modifying loci are expected to cause the breed-specific effect. Additional variants found on the wildtype haplotype surrounding the *VRTN* region in all sequenced Landrace animals point toward breed specific differences which are expected to be present also across the whole genome. This Landrace specific haplotype contains two missense mutations in the *ABCD4* gene, one of which is expected to have a negative effect on the protein function. Together, the integration of largescale genotype, phenotype and sequence data shows exemplarily how population parameters are influenced by underlying variation at the molecular level.

## Introduction

Number of teats (NTE) in pigs is a highly heritable trait and shows considerable variation across breeds (8–21) (Rohrer and Nonneman, 2017) and also within breeds (e.g., 12–20 in Landrace) (Lopes et al., 2014). Several genome-wide association studies (GWAS) have shown that NTE is a polygenic trait influenced by many different quantitative trait loci (QTL) scattered over nearly all chromosomes of the pig genome (Duijvesteijn et al., 2014; Lopes et al., 2014; Arakawa et al., 2015; Verardo et al., 2016; Yang et al., 2016; Tang et al., 2017; Uzzaman et al., 2018). Among these, a QTL on *Sus scrofa* chromosome 7 (SSC7) has been identified showing a large effect on NTE in several commercial breeding lines and crosses (e.g., Duijvesteijn et al., 2014; Rohrer and Nonneman, 2017; Dall’Olio et al., 2018). In the same region, a QTL was detected affecting number of ribs (RIB) (Mikawa et al., 2011). After fine mapping, Mikawa et al. (2011) identified a new transcript named Vertnin (*VRTN*) as being the most promising candidate gene. Several mutations have been detected in the region including a SNP in the promotor region (*g.19034A > C*) and an insertion of a PRE1-SINE element of 291-basepairs (*g.20311_20312ins291*) in the first intron of the *VRTN* gene (Mikawa et al., 2011; Ren et al., 2012; Fan et al., 2013). Just recently, *VRTN* has been characterized as a novel transcription factor affecting vertebra development (Duan et al., 2018). Duan et al. (2018) showed that these two mutations increase *VRTN* expression in an early embryonic stage in an additive way. Together these two mutations increase the number of thoracic vertebrae by 1 in the homozygous state (QQ). The effect of the PRE1-insertion on RIB has been validated in different commercial crossbred (Rohrer et al., 2015) and purebred lines (Dall’Olio et al., 2018). However, after correction for the PRE1 insertion allele, an additional negative effect on lumbar vertebrae was detected with a SNP located 500kb further proximal from *VRTN* and not in high linkage disequilibrium (LD) with the PRE1 insertion in crossbreds (Rohrer et al., 2015). Yang et al. (2016) also observed a pleiotropic effect of the insertion allele on number of vertebrae (NVE) and NTE in Chinese Erhualian, three European commercial purebred populations (Duroc, Landrace, Large White) and an intercross. However, a new candidate gene *LTBP2* (latent transforming growth factor binding protein 2) has been pinpointed by Zhang et al. (2016) for RIB in 596 Large-White x Chinese Minzhu F2 intercrosses where the *VRTN* insertion was not segregating but only the promoter SNP *g.19034A > C*. Finally, Park et al. (2017) describe a missense mutation in *LTBP2* at *c.4481A > C* associated with thoracic vertebrae number in 1,105 F2 animals of a Landrace cross with Korean native pigs where also the *VRTN* insertion is increasing thoracic vertebrae number independently.

In this study, we analyzed an extended data set of three commercial pig breeds linking genotype data of 20,366 Large White (LW), 23,398 Landrace (L), and 10,044 Duroc (D) animals with phenotypic data on NTE. In addition, NVE, and RIB data were scored from computer tomography (CT) images on 2,756 L and 2,961 D animals. We show that scoring phenotypes closer to the molecular basis of the observed variation (NVE or RIB vs. NTE) increases population genetic parameters such as heritability and explained genetic variance considerably. Furthermore, a detailed analysis combining medium and high density SNP data with whole-genome sequence (WGS) data with functional parameters of mutations has been performed. Our results show that the two functional mutations analyzed by Duan et al. (2018) are in high LD with the most significant SNPs from the GWAS studies in all breeds, however, the phenotypic effect on NVE depends on the genetic background.

## Materials and Methods

### Data

In this study, data from the three pig populations LW (Large White-based), L (Landrace) and D (Duroc) were evaluated. The LW population was located in Dutch nucleus farms and were born between 2006 and 2017 (data obtained by Topigs Norsvin, the Netherlands). The Norwegian L and D populations were located in Norwegian nucleus farms and at a boar testing station (Hamar, Norway) and were born between 2010 and 2017 (data obtained by Norsvin, Norway).

**FIGURE 1:**
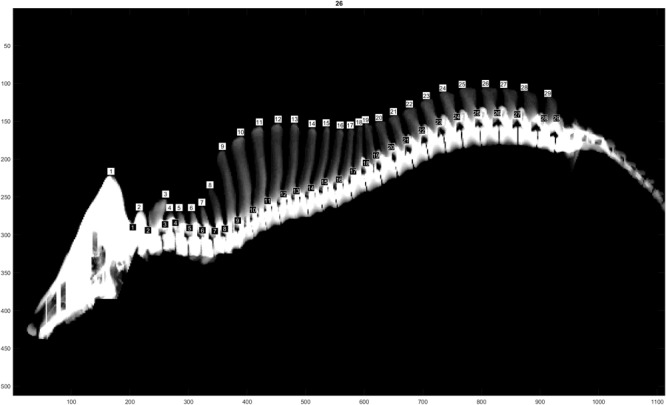
Computer tomography image illustrating the NVE recording.

The traits evaluated were NTE, recorded at birth on both males and females from all three populations, and NVE and RIB retrieved from CT images only on males from the L and D populations at 120 Kg of live weight. The traits NVE and RIB were recorded using the CT scanner GE Healthcare LightSpeed 32 VCT, and the settings used were 120 kV, slice thickness 1.25 mm and dynamic mA (400–500 mA) adjusting for object thickness. Prior to scanning, the boars were sedated using Azaperone (Stresnil Vet^®^, Janssen-Cilag Ltd., Buckinghamshire, United Kingdom), which was injected intramuscularly. The whole skeleton was segmented out by applying a threshold value at 200 Hounsfield units to the full CT volumes. Individual vertebras and ribs were segmented and identified in accordance with Gangsei and Kongsro (2016). Furthermore, a visual count of the total number of vertebras was performed (Figure 1). This number includes the total number of cervical, thoracic and lumbar vertebras, omitting sacrum and coccyx vertebras. In pigs, the number of cervical vertebras is fixed at 7, whereas the number of thoracic and lumbar vertebras might vary (King and Roberts, 1960). The trait RIB was counted on both right and left sides of each animal. However, due to the similarity of the results when analyzing right and left RIB separately, in this study we will show only the results of the analyses using RIB from the right hand side.

For each trait, two datasets from each population were used: ALL and GENOTYPED (See Table 1 for descriptive statistics). The dataset ALL consisted of all genotyped animals and their contemporaries that had phenotypes (275,513 LW, 313,475 L, and 12,672 D for NTE, 2,756 L and 2,961 D for NVE, and 2,653 L and 2,874 D for RIB). Using ALL, the phenotypes were pre-corrected for all non-genetic effects. The pre-corrected phenotype was used as the response variable in further analysis. The non-genetic effects were estimated by the pedigree-based linear models 1 (NTE) and 2 (NVE and RIB) in ASReml v3.0 (Gilmour et al., 2009):

**Table 1:** Summary statistics.

Trait^a^	Population	Dataset^b^	N^c^	Mean	SD^d^
NTE	Large white	ALL	275,513	15.30	1.08
		GENOTYPED	20,366	15.73	1.02
	Landrace	ALL	313,475	15.84	1.03
		GENOTYPED	23,398	15.99	1.04
	Duroc	ALL	12,672	12.93	1.05
		GENOTYPED	10,044	12.93	1.04
NVE	Landrace	ALL	2,756	29.78	0.53
		GENOTYPED	1,873	29.75	0.54
	Duroc	ALL	2,961	28.72	0.60
		GENOTYPED	2,384	28.71	0.60
RIB	Landrace	ALL	2,653	15.47	0.71
		GENOTYPED	1,802	15.48	0.71
	Duroc	ALL	2,874	14.57	0.62
		GENOTYPED	2,322	14.57	0.61

*^*a*^NTE, number of teats; NVE, total number of vertebrae; RIB, number of ribs on the right side of the animal. ^*b*^ALL, the whole population used in the pre-adjustment of the phenotypes, which includes the animals from GENOTYPED and their contemporaries; GENOTYPED, genotyped and phenotyped animals used for the GWAS. ^*c*^N, number of animals. ^*d*^SD, standard deviation of the traits in each dataset of each population.*

(1) $$\begin{array}{c} NTE_{\mathrm{ijkl}}=\mu +sex_{i}+hy_{j}+a_{k}+litter_{l}+e_{\mathrm{ijkl}} \end{array}$$

where NTE*_ijkl_* was the NTE of the *k* animal; μ is the overall mean, sex*_i_* was the fixed effect of sex *i*, hy*_j_* was the fixed effect of the herd-year *j* of birth, a*_k_* was the random additive genetic effect of animal *k*, litter*_l_* was the random effect of litter *l* and e*_ijkl_* was the random residual effect. The vector of additive genetic effects was assumed to be distributed as ∼*N*(**0,A**$\sigma_{a}^{2}$), which accounted for the (co)variances between animals due to relationships by formation of an **A** matrix (pedigree-based numerator relationship matrix), $\sigma_{a}^{2}$ being the additive genetic variance. The vector of litter effects was assumed to be distributed as ∼*N*(**0,I**$\sigma_{l}^{2}$), with **I** being an identity matrix and $\sigma_{l}^{2}$ the litter variance. The vector of residual effects was assumed to be distributed as ∼*N*(**0,I**$\sigma_{e}^{2}$), $\sigma_{e}^{2}$ being the residual variance.

(2) $$\begin{array}{c} {NVE/RIB}_{\mathrm{ijk}}={\mu +\mathrm{year}}_{i}{+\mathrm{farm}}_{j}{+a}_{k}{+e}_{\mathrm{ijk}} \end{array}$$

where NVE/RIB*_ijk_* was either the number vertebrae or ribs of the *k* animal; μ is the overall mean, year*_i_* was the fixed effect of year *I* of birth, farm*_j_* was the fixed effect of the herd *j* of birth, a*_k_* and e*_ijkl_* was as described above for model (1).

The dataset GENOTYPED was a subset of ALL consisting of all animals that had both phenotypes and genotypes (20,366 LW, 23,398 L, and 10,044 D for NTE, 1,873 L and 2,384 D for NVE, and 1,802 L and 2,322 D for RIB). This dataset was used to perform the GWAS.

### Medium Density Genotypes

All animals from the GENOTYPED dataset were genotyped using a medium density SNP chip. Genotyping was performed at CIGENE (University of Life Sciences, Ås, Norway) and at GeneSeek (Lincoln, NE, United States), mainly using the (Illumina) GeneSeek custom 80K SNP chip (Lincoln, NE, United States). However, a small part of the animals from the three populations were genotyped using the (Illumina) GeneSeek custom 50K SNP chip (Lincoln, NE, United States) and the Illumina Porcine SNP60 Beadchip (Illumina, San Diego, CA, United States).

Quality control consisted of excluding SNPs with GenCall < 0.15 (Illumina Inc., 2005), call rate < 0.95, minor allele frequency < 0.01, strong deviation from Hardy-Weinberg equilibrium (χ^2^> 600), SNPs located on sex chromosomes and unmapped SNPs. The positions of the SNPs were based on the Sscrofa11.1 assembly of the reference genome. Animals with frequency of missing genotypes ≥ 0.05 would be removed from the dataset. However, all genotyped animals had a frequency of missing genotypes < 0.05 and were therefore kept for further analyses.

After quality control, the remaining missing genotypes of the animals genotyped with the (Illumina) GeneSeek custom 80K SNP chip were imputed within population using Fimpute v2.2 (Sargolzaei et al., 2014). At the same time, the animals genotyped with the other two chips had their genotypes imputed to the set of SNPs on the (Illumina) GeneSeek custom 80K SNP chip that passed the quality control. After quality control and imputation, 50,717 SNPs for LW, 44,961 SNPs for L and 43,309 SNPs for D were available and were used in the imputation toward the high density SNP chip.

### High Density Genotypes

Genotyping of high density genotypes was also performed at CIGENE (University of Life Sciences, Ås, Norway) and GeneSeek (Lincoln, NE, United States). In total, 290 LW, 415 L, and 140 D animals from the GENOTYPED dataset were in addition genotyped using the Axiom porcine 660K array from Affymetrix (Affymetrix Inc., Santa Clara, CA, United States). These animals were the most influential sires from each population (sires with the largest number of offspring in the GENOTYPED dataset). Quality control of 660K array was as described above for the medium density genotypes. The imputation from 80K genotypes toward 660K genotypes was performed within population using Fimpute v2.2 (Sargolzaei et al., 2014). After quality control and imputation, there were 527,186 SNPs for LW, 462,414 SNPs for L and 441,288 SNPs for D, which were used in the GWAS.

### Haplotype Analysis and Recombinant Identification

Beagle v.4.1 (Browning and Browning, 2016) was used to phase the medium and high density genotype data. Bcftools v1.5-28-ge9ec882 (Li et al., 2009) was used to extract the phased genotypes in the QTL region SSC7: 97–98 Mb. Next, we constructed haplotypes in the region SSC7: 97–98 Mb using PyVCF (Casbon, 2012) and report recombinant animals for animals that carry a different haplotype compared to the parent animals.

### Sequence Data

Analysis of WGS data was done to construct a sequence level SNP dataset for the QTL region (SSC7: 85–105 Mb) in L and D. WGS data from 24 L and 23 D boars were available for this purpose. The boars were previously frequently used AI boars and all of them were part of the GENOTYPED dataset. DNA extraction and the sequencing procedure for whole genome re-sequencing is described in detail in van Son et al. (2017). The reads were 2 × 100 basepair paired-end reads and mapped to *Sus scrofa* build 11.1, duplicated marked and indexed using the speedseq align module available in SpeedSeq (Chiang et al., 2015). Freebayes v.1.3.1 (Garrison and Marth, 2012) was used to detect variants in the QTL region (92–103 Mb), using a minimum alternate allele count of 2, and identified 179,241 and 190,260 putative variants in L and D, respectively. Filtering of variants was done by VCFtools v.0.1.14 (Danecek et al., 2011) and SAMtools bcftools v.1.3.1 (Li et al., 2009). The filtering criteria were that both reference and alternate allele must be present on both strands, a minimum quality score of 25, a mapping quality of > 10 for both alleles at a SNP position and a sequencing depth > 6 and < 2000. Variants with more than one unique non-reference allele were removed for imputation purposes and a distance of at least 4 and 10 basepairs to the next insertion/deletion was applied for SNPs and indels, respectively. This resulted in a total of 80,392 and 89,725 high quality SNPs available for imputation in L and D, respectively. Newly detected SNPs have been deposited to EVA with accession number PRJEB27233.

### Imputation to Sequence

The WGS data SNPs from the 24 L and 23 D boars in the SSC7 QTL region were phased within breed using Beagle v.4.1 (Browning and Browning, 2016). Prior to imputation, the 660K array SNPs described in “high density genotypes” were compared with the WGS data SNPs using conform-gt (Browning and Browning, 2016) to remove array SNPs that were not present in the WGS data and to adjust corresponding SNPs to match chromosome strand and allele order. In L, 954 of the 4189 array SNPs in the QTL region were removed by conform-gt because they were not in the reference dataset or because the chromosome strand was unknown, whereas in D, 744 of 4539 SNPs were removed. The rest of the 660K array SNPs were included for imputation to sequence level using Beagle v.4.1 and default settings.

### GWAS

A single-SNP GWAS was performed with the GENOTYPED dataset within each population using the following linear animal model in GCTA (Yang et al., 2011, 2014):

(3) $$\begin{array}{c} y^{*}{}_{k}=\mu +X\hat{\beta }+u_{k}+e_{k} \end{array}$$

where y^∗^*_k_* was the pre-corrected phenotype of the *k* animal (pre-corrected for all non-genetic effects, as explained in section Data); μ the average of the pre-corrected phenotype; *X* was the genotype (0, 1, 2) of the *k* animal for the evaluated SNP; $\hat{\beta }$ was the unknown allele substitution effect of the evaluated SNP; u*_k_* was the random additive genetic effect, being that the vector of additive genetic effects was assumed to be distributed as ∼*N*(**0,G**$\sigma_{a}^{2}$), which accounted for the (co)variances between animals due to relationships by formation of an **G** matrix (genomic numerator relationship matrix build using the imputed 660K genotypes), $\sigma_{a}^{2}$ being the additive genetic variance; and e*_k_* was the random residual effect which was assumed to be distributed as ∼*N*(**0,I**$\sigma_{e}^{2}$).

The genetic variance explained by a SNP ($\sigma_{\mathrm{snp}}^{2}$ = *2pqα^2^*) was estimated based on the allele frequencies (*p* and *q*) and the estimated allele substitution effect (*α*). The proportion of phenotypic variance explained by the SNP was defined as $\sigma_{\mathrm{rmsnp}}^{2}$/$\sigma_{p}^{2}$, where $\sigma_{p}^{2}$ is total phenotypic variance (sum of the additive and residual variances) which was estimated based on model (3) without a SNP effect. Significant SNPs and QTL were detected using a *p*-value < 1.0 × 10^-8^.

After the GWAS using the imputed 660K data, we extracted all SNPs from the imputed WGS data that are located 5 Mb upstream and downstream the most significant 660K SNP for the traits NVE and RIB. With this data, we performed WGS association analyses for NVE and RIB aiming to identify stronger association with these phenotypes. These analyses were also performed applying model (3) in GCTA (Yang et al., 2011, 2014). LD as measured by *r*^2^ was calculated between SNPs using Plink 1.9 (Purcell et al., 2007).

### Identification of Functional Variants From Sequencing Data

The variants identified by WGS data analysis were used to find potentially functional mutations. All L and D animals with WGS and NVE data (34 animals) were grouped based on their genotype for the *VRTN* insertion *g.20311_20312ins291*. Three groups were created: Homozygous Wild Type (7 animals) (wt/wt), Heterozygous Insertion (18 animals) (wt/ins), and Homozygous Insertion (9 animals) (ins/ins). The filtered SNPs generated with sequence data was then used to search for variants that were overrepresented in the different groups.

Variant calls for all samples were annotated with the Ensembl variant effect predictor (McLaren et al., 2016), version 90.6, using Ensembl release 90 and *Sus scrofa* genome build 11.1. Known regulatory regions of the human *LTBP2* gene (Davis et al., 2014) were mapped from GRCh37 to GRCh38, using the UCSC liftOver tool (Kent et al., 2002). These mapped coordinates were used as input for the Ensembl comparative genomics tool to identify the corresponding regions in build 11.1 of the *Sus scrofa* genome (Zerbino et al., 2018). The corresponding pig genome sequences were then used as input to EMBOSS Needle to generate local alignments and percentage identities for the promoters (Rice et al., 2000).

### Genotyping of *VRTN* Insertion Variant

The 291-basepair insertion *g.20311_20312ins291* (GenBank accession number AB554652, position 7:97615880), was genotyped using primers and PCR conditions reported by Yang et al. (2016). The PCR products were separated by 2% agarose gel electrophoresis and the genotypes were visually recorded by inspection of amplicon length. The insertion allele was represented by a 411-basepair amplicon whereas the wild type allele was 120 basepairs. Ninety six animals were genotyped for the *VRTN* insertion, out of which 82 had phenotypes and the rest of the animals were parents and grandparents used to confirm observed genotypes.

Visualization of the genomic region containing the *VRTN* insertion was done in the WGS animals by the Integrative Genomics Viewer (IGV) software (Robinson et al., 2011; Thorvaldsdóttir et al., 2012). This allowed us to genotype the subset of 18/15 L/D animals with NVE phenotypes using their sequence data (see Supplementary Figure S1 for examples on how this was done). Only animals showing at least two forward and two reverse reads for the insertion and the wildtype allele were genotyped as heterozygous (wt/ins). For homozygous wt/wt or ins/ins genotypes, only animals with at least 5 reads covering the insertion point were included. The insertion is supported by both split-reads and discordantly mapped pairs (Supplementary Figure S1) as well as reduced coverage of aligned sequences. Genotypes of WGS animals were also derived by IGV for the SNP promoter mutation in *VRTN* (*g.19034A > C, rs709317845*, position 7:97614602) (Fan et al., 2013) and the missense mutation in *LTBP2* (*c.4481A > C, rs322260921*, position 7:97751432) (Park et al., 2017).

## Results

**FIGURE 2:**
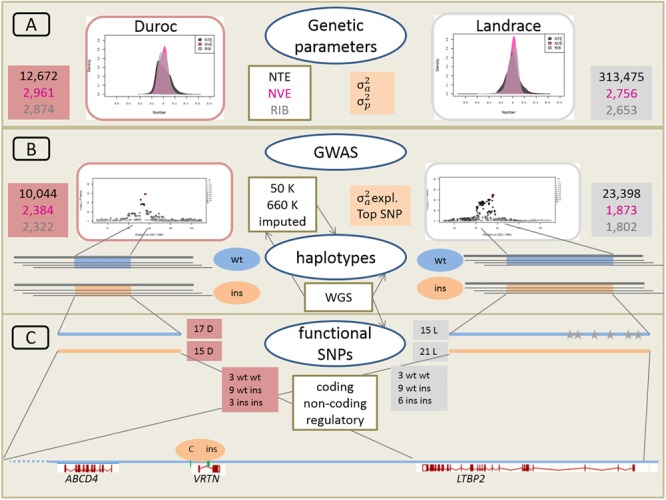
Schematic overveiw of the approach linking large-scale phenotypic, genetic, and genomic data. **(A)** Phenotypic distribution and estimation of additive genetic variance (σ^2^_a_) and phenotypic variance (σ^2^_p_) for Duroc and Landrace breeds for NTE, number of vertebrae (NVE), and number of ribs (RIB). Number of animals for each population and each trait is indicated in red and gray boxes for Duroc and Landrace, respectively. **(B)** Genome wide association analyses (GWAS) with medium density (50K) and imputed high density (660K) SNP sets as well as imputed from whole genome sequence (WGS) data. Number of animals with 50K data in red and gray boxes. Estimation of haplotypes from 660K SNP data and search for identical core haplotype associated with two functional variants (ins) in the Vertnin (*VRTN*) gene compared to the wildtype (wt) haplotypes. **(C)** Search for potentially functional SNPs and indels in coding (missense, non-sense) and non-coding (splice sites) regions in the core haplotypes of 16 D (3 wt wt, 9 wt ins, 3 ins ins) and 18 L (3 wt wt, 9 wt ins, 6 ins ins) animals for the wt and ins alleles. Mapping of regulatory enhancer sequences in *LTBP2* from human genome. Identification of SNP and indel variants only present on each of the 15 wt haplotype in L breed indicated as gray stars. Underneath annotation of the relevant candidate genes from *Sus scrofa* reference genome build 11.1, and position of the two causative variants in the *VRTN* gene.

A schematic overview of the approach linking large-scale phenotypic, genetic and genomic data is given in Figure 2.

### Heritabilities and GWAS

Table 1 shows the descriptive statistics for all traits and breeds. The mean value for NTE is around 3 teats higher in the LW and L breeds compared to D (16 vs. 13 for genotyped animals). The mean NVE and RIB is 1 unit higher in the L breed than in D (29.8 vs. 28.7 and 15.5 vs. 14.6, respectively).

The heritability (h^2^), defined as the proportion of the total phenotypic variance explained by additive genetic variance, for NTE was 0.41 (LW), 0.39 (L), and slightly lower for the D breed (0.28) (Table 2). For NVE, the h^2^ was considerably greater than the h^2^ for NTE, being 0.59 in the L breed and 0.62 in the D breed. The h^2^ for RIB, compared to the h^2^ for NTE, was even greater (0.78) in the D breed, however, it was considerably lower (0.24) in the L breed (Table 2). Data on NVE and RIB were not available from the LW breed.

**Table 2:** Heritability h^2^ and parameters for the most significant SNP for each trait in each breed from the GWAS.

Breed	Trait ^a^	h^2b^	SNP	SSC7^c^	-Log_10_(*P*)	Freq.^d^	Effect^e^	SD^f^	Var._exp_^g^
LW	NTE	0.41	AX-116757987	97.57	74	0.23	0.38	0.02	0.05
L	NTE	0.39	AX-116329721	97.62	50	0.70	0.33	0.02	0.05
	NVE	0.59	AX-116329717	97.53	84	0.69	0.49	0.03	0.34
	RIB	0.24	AX-116329717	97.53	54	0.69	0.49	0.03	0.20
D	NTE	0.28	AX-116777212	97.61	59	0.33	0.38	0.02	0.06
	NVE	0.62	AX-116329719	97.59	184	0.31	0.68	0.02	0.52
	RIB	0.78	AX-116329688	97.60	260	0.31	0.83	0.02	0.69

*^*a*^NTE, number of teats; NVE, total number of vertebrae; RIB, number of ribs on the right side of the animal. ^*b*^h^2^, heritability. ^*c*^SSC7, position in Mb on chromosome 7. ^*d*^Freq., frequency of the allele increasing NTE, NVE, or RIB. ^*e*^Effect, allele substitution effect. ^*f*^SD, standard deviation of the allele susbtitution effect. ^*g*^Var._*exp*_, percentage total phenotypic variance explained by the most significant SNP.*

**FIGURE 3:**
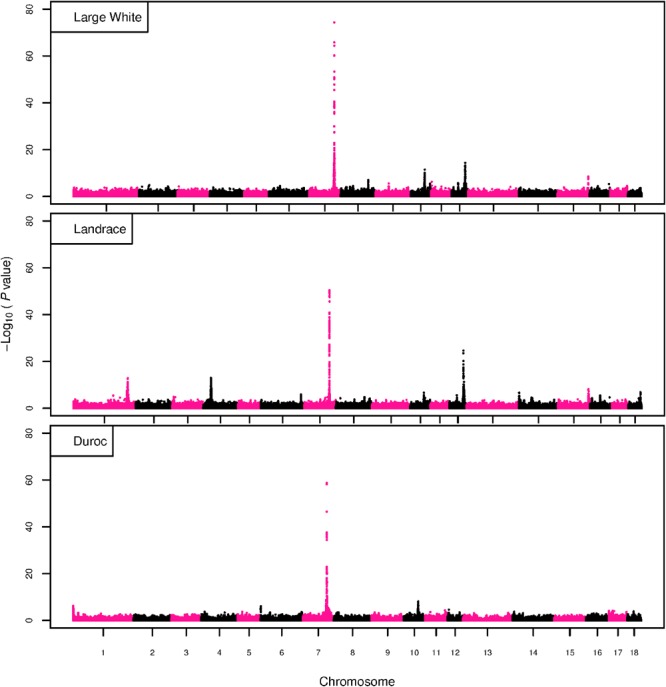
GWAS plot for NTE with imputed 660K SNP chip data. On the *y*-axis is the –log_10_(*p*-values) of single SNP association with NTE in LW, L, and D breeds. On the *x*-axis is the physical position of the SNP across the 18 autosomes.

**FIGURE 4:**
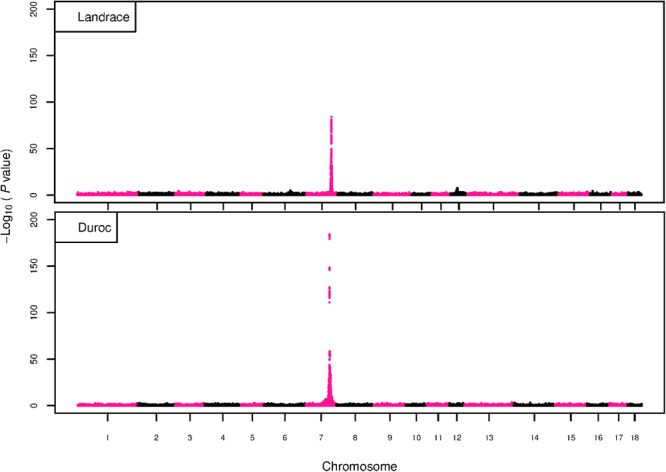
GWAS plot for total NVE with imputed 660K SNP chip data. On the *y*-axis is the –log_10_(*p*-values) of single SNP association with NVE in L and D breeds. On the *x*-axis is the physical position of the SNP across the 18 autosomes.

**FIGURE 5:**
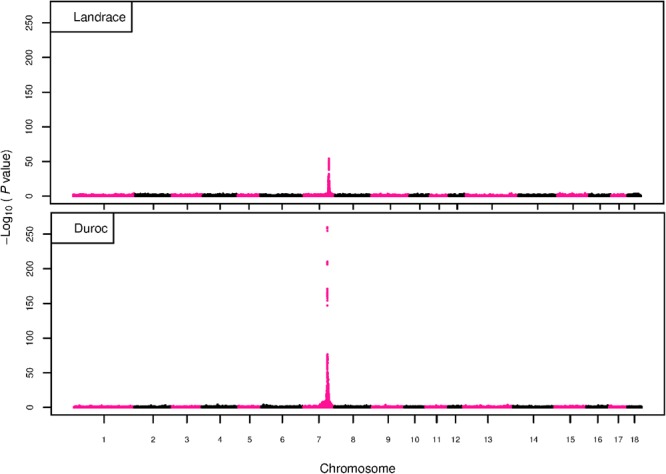
GWAS plot for RIB with imputed 660K SNP chip data. On the *y*-axis is the –log_10_(*p*-values) of single SNP association with RIB in L and D breeds. On the *x*-axis is the physical position of the SNP across the 18 autosomes.

**FIGURE 6:**
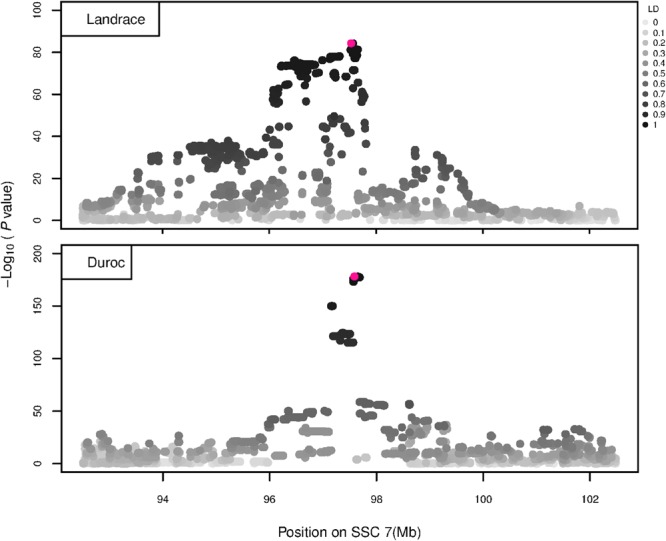
GWAS plot for NVE using imputed whole-sequence data. On the *y*-axis is the –log_10_(*p*-values) of single SNP association with NVE in L and D breeds. On the *x*-axis is the physical position of the SNP in the SSC7 QTL region. Linkage disequilibrium (LD) is given in a scale of 0–1 as a measure of the pairwise correlation between the most significant SNP (pink dot) and all other SNPs.

The GWAS results for NTE show a strong QTL on SSC7 in all three breeds in the same region (Figure 3). In addition, other QTL segregate on several other chromosomes, especially in the LW and L breeds. Some QTL regions overlap between the three populations but a few QTL are breed specific (Table 3 and Supplementary File S1). All these additional QTL disappear in the L and D breeds in the GWAS for the traits NVE and RIB (Figure 4, 5 and Supplementary File S1). Only a strong significant peak remains for SSC7 in the same region for the L and D breeds (Table 2). The position of the most significant SNP (top SNP) differs slightly between traits and populations around 97.6 Mb on SSC7 (build 11.1). Only in the L breed, the top SNP shifts around 90 kb from 97.62 Mb for NTE (*AX-116329721*) to 97.53 Mb for NVE and RIB (*AX-116329717*). However, Figure 6, 7 show that the extent of LD between the top SNP and all other SNPs in the QTL region is much larger in the L breed than in D for both NVE and RIB. For all GWAS, the most significant SNP from the imputed 660K SNP set is also the top SNP from the imputed WGS association analyses (Supplementary File S2). The frequency of the allele of the top SNP that is related to increased NTE is more than twice as high in the L breed compared to D (0.70 vs. 0.33) and the lowest in LW (0.23) (Table 2). For NVE and RIB the allele frequency values in the L and D breeds are only slightly lower than for NTE (0.69 vs. 0.31). However, the significance levels differ remarkably between traits and breeds. The –log_10_ of the *p*-value increases from 50 to 84 in the L breed and from 59 to 184 in D for NTE and NVE, respectively. Furthermore, scoring RIB instead of NVE increases significance levels considerably further but only in D and decreases extremely in the L breed (260 vs. 54, respectively). This is in line with the explained variance increasing from 5 and 6% for NTE up to 34% (L) and 52% (D) for NVE. However, for RIB, we observe a further increase of explained variance up to 69% in D whereas a severe reduction is seen again in the L breed down to 20%. The size of the effect for NTE is comparable across breeds between 0.33 (L) and 0.38 (LW and D). For NVE the effect is much higher in D than in L (0.68 vs. 0.49) and for RIB it only increases in D up to 0.83 but remains the same in the L breed (0.49).

**Table 3:** Genomic regions associated with NTE in LW, L, and D populations.

				topSNP		Gene/
			# Significant	location	topSNP	nearest
Breed	SSC	Position (Mb)	SNPs	(Mb)	*p*-value	gene
Large white	7	93.7–99.2	195	97.57	4.68e-75	*VRTN*
	10	47.2–47.9	13	47.88	3.30e-12	*FRMD4A*
	12	50.4–50.6	23	50.60	4.02e-15	*SMTNL2*
	15	134.8–134.9	11	134.80	3.27e-09	*ARL4C*
Landrace	1	23.9–24.1	25	24.05	1.55e-13	*ANKS6*
	4	29.0–30.9	57	29.21	1.03e-13	*EIF3E*
	7	95.9–97.8	147	97.62	4.13e-51	*VRTN*
	12	50.3–52.6	64	50.36	6.11e-10	*UBE2G1*
	15	134.62–134.64	2	134.64	6.59e-09	*ARL4C*
Duroc	7	82.8–99.3	261	97.61	1.69e-59	*VRTN*
	10	47.8–47.9	7	47.88	7.55e-09	*FRMD4A*

*The QTL regions for NTE are presented with positions (build 11.1), number of significant SNPs (p-value < 1.0e-08), topSNP location and p-value, and the gene/nearest gene of the topSNP.*

**FIGURE 7:**
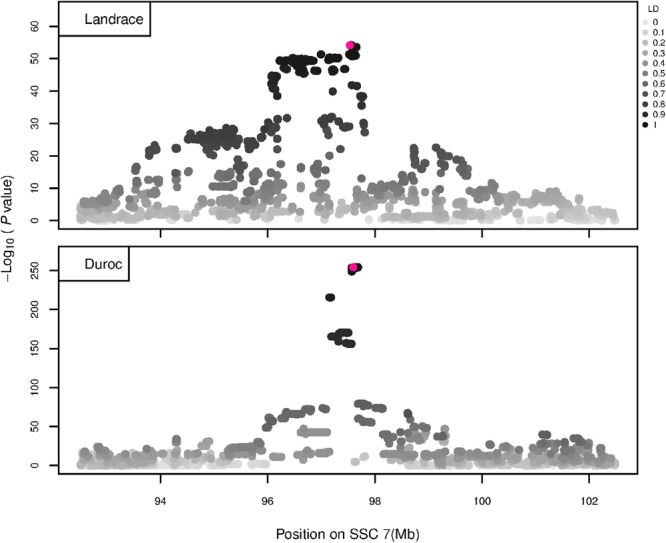
GWAS plot for RIB using imputed whole-sequence data. On the *y*-axis is the –log_10_(*p*-values) of single SNP association with RIB in L and D breeds. On the *x*-axis is the physical position of the SNP in the SSC7 QTL region. Linkage disequilibrium (LD) is given in a scale of 0–1 as a measure of the pairwise correlation between the most significant SNP (pink dot) and all other SNPs.

### Sequencing Data Analysis

The previously identified *VRTN* insertion (*g.20311_20312ins291*) (Mikawa et al., 2011) and promoter SNP (*g.19034A > C, rs709317845*) (Fan et al., 2013) were compared to NVE in the WGS animals and some relatives by PCR and IGV (*n* = 96). Pigs with the insertion allele and the C allele of the promoter SNP were associated with increasing NVE in both L and D breeds, and the two variants were completely linked in the examined animals. The PCR genotyping of the insertion confirmed the findings by IGV.

When grouping animals by *VRTN* genotypes and analyzing the WGS data within the SSC7 QTL region for protein coding variants, no other compelling candidates for functional coding variants were found (Supplementary File S3, Table X7). Protein coding variants in this region occur either in far too many individuals or far too few individuals to be having an impact on the observed phenotypes (Supplementary File S3, Tables X3–X5 show variants unique to groups, and Supplementary File S3, Table X6 shows variants shared between groups). However, there were large differences between the three groups for some non-coding variants, affecting several genes (Supplementary File S3, Tables X2–X6). For example, multiple mutations were identified in ATP binding cassette subfamily D member 4 (*ABCD4*) that are far more common in the wt/wt and wt/ins samples, with *ABCD4* having the most unique mutations of any gene in this locus. Several non-coding mutations in ABCD4 occur in 7/7 of the wt/wt samples and 18/18 wt/ins samples, and 0/9 of the ins/ins samples (Table 4 and Supplementary File S3, Table X6). These non-coding variants could have functional effects that impact NVE, but without transcriptomics data, and with poor functional annotation of the non-coding regions in this locus, it is not possible to determine which of these variants could have a functional impact.

**Table 4:** Identified variants on the same haplotype as *VRTN* insertion and promoter SNP.

Variant	Chromosome	Position	Ref/Alt	Consequence	Gene Symbol	rsID	LD D	LD L
7_97563673_T/G	7	97563673	T/G	Downstream	ABCD4	–	0.693193	0.970185
7_97568605_T/A	7	97568605	T/A	Intronic	ABCD4	rs711873120	0.989868	0.888563
7_97568606_A/C	7	97568606	A/C	Intronic	ABCD4	rs699009491	0.989868	0.888563
7_97568835_G/A	7	97568835	G/A	Intronic	ABCD4	rs692051374	0.989868	0.888563
7_97569136_C/T	7	97569136	C/T	Intronic	ABCD4	WU_10_2_7_103412699	0.993029	0.861442
7_97571221_C/T	7	97571221	C/T	Intronic	ABCD4	–	0.989868	0.888563
7_97571322_A/T	7	97571322	A/T	Intronic	ABCD4	–	0.989868	0.888563
7_97571384_CAGC/CGG	7	97571384	CAGC/CGG	Intronic	ABCD4	–	0.989868	0.888563
7_97572779_G/A	7	97572779	G/A	Intronic	ABCD4	–	0.989868	0.888563
7_97572788_C/T	7	97572788	C/T	Intronic	ABCD4	–	0.989868	0.888563
7_97574280_CG/CAG	7	97574280	CG/CAG	Intronic	ABCD4	–	0.989868	0.888563
7_97579254_T/C	7	97579254	T/C	Intronic	ABCD4	rs1112162366	0.994818	0.888563
7_97579520_G/T	7	97579520	G/T	Intronic	ABCD4	–	0.994818	0.888563
7_97606621_A/T	7	97606621	A/T	Intergenic	ABCD4-VRTN	rs331843703	1	1
7_97642098_C/G	7	97642098	C/G	Intergenic	VRTN-SYNDIG1L	rs337650751	0.996804	0.980266

*At these identified variants, the animals (n = 34) with VRTN homozygous ins/ins (ins/ins at the VRTN insertion g.20311_20312ins291, position 7:97615880, and CC at the VRTN promoter SNP g.19034A > C, position 97614602) and wt/wt have opposing homozygous genotypes and all the Qq animals have heterozygous genotypes. LD as r^2^-values is calculated with respect to the two VRTN mutations in Duroc (D) and Landrace (L). More information can be found in Supplementary File S3, Table X6.*

### Haplotype Analysis and Recombinant Identification

We determined the haplotypes that are associated with the two functional *VRTN* mutations and find a single relatively high frequency haplotype associated within each breed. However, several haplotypes at lower frequency are also associated with the two functional *VRTN* mutations; an example of the associated 80K and 660K haplotypes in the D breed is presented in Supplementary File S4. They all have a core haplotype of seven SNPs in common surrounding the two *VRTN* variants.

Duan et al. (2018) showed that the promoter variant and the PRE1 insertion increase *VRTN* expression in an additive way. To estimate the effect of both causal mutations separately we searched the genotype data for recombinant animals. Interestingly, we identified two sequenced recombinant animals, but only within the LW breed. One animal is wt/wt for the promoter SNP, while being heterozygous for the *VRTN* insertion. The other animal is heterozygous for the promoter SNP, while being homozygous for the *VRTN* insertion (Supplementary Figure S2). Haplotype analysis (on medium density SNP chip) confirmed that both animals carry a separate recombinant haplotype. The haplotypes are segregating with a combined frequency of about 1.8% in LW. Unfortunately, no phenotypic data on NVE and RIB are available for this breed.

Haplotype analysis also identified a haplotype that is specific for the L breed animals not carrying the *VRTN* insertion allele (Supplementary File S5, Table S1). The haplotype was not present in D or in L ins/ins animals and might explain the different effect on RIB found in the L breed. There are two missense mutations within this haplotype, both located in *ABCD4*, with SIFT values of 0.03 and 0.1. Moreover, two splice region variants, located in *ABCD4* and *VSX2* (visual system homeobox 2), are putative causal candidates within this wildtype haplotype observed only in L. The SNPs located on the 660K chip that are segregating with this haplotype are presented in Supplementary File S5, Table X2.

### LTBP2 Regulatory Region Analysis

Park et al. (2017) describe an effect on number of thoracic vertebrae further downstream of *VRTN* and pinpoint *LTBP2* as a candidate gene. Here, known human *LTBP2* regulatory regions (Davis et al., 2014) were mapped to the corresponding regions of the pig genome. The majority of these regions were found to have a high sequence identity between the species, indicating that they may perform similar regulatory functions in the pig genome (Supplementary File S3). Additionally, the previously reported *LTBP2* variant (*c.4481A > C, rs322260921*, position 7:97751432) (Park et al., 2017) was investigated using sequenced animals. The *LTBP2* variant showed no effect when sorting animals by the *VRTN* genotype (Supplementary File S3, Tables X6, X7). Moreover, the SNP variant in the *LTBP2* gene is not in complete LD with *VRTN* variants in our pig breeds (*r*^2^ of 0.70 and 0.44 in L and D, respectively) and it is outside the core region identified in haplotype analyses (Supplementary File S4). This makes it unlikely that the insertion is directly affecting *LTBP2* regulation, but it is possible that pig *LTBP2* has additional regulatory regions compared to the human version, and these could be affected by the insertion.

## Discussion

### Parameters of Genetic Variation at the Population Level

In a previous study, we identified a QTL for NTE on SSC7 in a population of 936 LW animals (Duijvesteijn et al., 2014). Expanding the data to 2,620 individuals of the same LW population and adding 6,090 and 3,798 animals of the two other purebred breeds also evaluated in this study (L and D, respectively), we identified the same QTL segregating in all three populations (Lopes et al., 2017b). In the current study, we expanded the data even further to more than 20,000 LW and L animals and more than 10,000 D animals, reconfirming the QTL region for NTE on SSC7.

In this study, we show that the size of the effect is comparable in all three breeds, but the frequency of the allele related to increased NTE at the top SNP is more than twice as high in L as in LW and D breeds (Table 2). Assuming that the underlying mutation was affecting NVE (Duijvesteijn et al., 2014; Rohrer et al., 2015), we used detailed phenotypes for the vertebral column available for the L and D breeds from CT scan data. Indeed, examining the phenotype closer to the causative variation increased heritability, that is explaining an additional 20% (L)–34% (D) of the phenotypic variation by additive genetic effects from just one QTL. Noise from other QTL segregating for NTE is not relevant for NVE, as can be seen from the GWAS results (Figure 4). With only 24% of the animals available for NVE in D and 8% in L, compared to the number of animals available for NTE in these breeds, a much higher significance of the effect is obtained. Moreover, the size of the effect is much higher because additional variation of loci further downstream in the developmental cascade of teat development (Veltmaat, 2017) is not diluting the genetic effect on NVE. A further reduction in noise by counting RIB was expected because this QTL has been reported to affect thoracic vertebrae only and domestic pig populations also show variability in number of lumbar vertebrae (Rohrer et al., 2015), which was included in the NVE count. However, a further increase in effect size and h^2^ for RIB compared to NVE was only observed in D, whereas a strong reduction of h^2^ was estimated in L. The size of the effect on RIB remained the same (0.49) for NVE in L and in the expected range as reported for thoracic vertebrae (ribs) by Duan et al. (2018), with the homozygous QQ animals (double mutant allele, insertion and promoter SNP together) having 1 vertebra more. Apparently, the effect of this QTL is disturbed by other genetic variation affecting rib development in L, which is not present in the D breed. Breed-specific effects have been reported earlier for other traits in pigs (Lopes et al., 2017a; Sevillano et al., 2018). However, it is also important to highlight that the allele related to increased RIB in the L breed is going toward fixation, decreasing the genetic variability in this population and therefore could be responsible for the lower h^2^ and total phenotypic variance for RIB in L compared to D. Furthermore, the size of the allelic effect in D is much larger (0.68 NVE and 0.83 RIB) generating 1.3 vertebrae more in the homozygous state compared to homozygous wildtype animals and even developing 1.6 more ribs phenotypically. The sum of these effects together (allele frequency and h^2^) is mirrored by the phenotypic variance explained by the top SNP which accumulates to 69% in D for RIB and is the main indicator for the expected breeding progress in this trait.

To examine whether other loci close to *VRTN* had an effect on NTE, NVE, or RIB, as previously reported by Rohrer et al. (2015), the most significant SNP was included as a fixed effect in the model. Correction for the top SNP showed that no other genetic variation is present in our populations for either of the traits. Although our GWAS results indicate that the traits NVE and RIB are controlled by one QTL of large effect, the top SNP does not seem to be the causal mutation. The variance explained by the top SNP was only up to 88% of h^2^ for these traits in these breeds. Therefore, there is still genetic variance that is not captured by this SNP. Trying to get closer to the causal mutation, we also performed a GWAS using imputed WGS data. However, no additional information was obtained from increasing the resolution to imputed WGS variants as the same top SNP was identified using both SNP chip and imputed WGS data. Although the added benefit of WGS data is including the functional mutation, the WGS dataset mostly contains haplotypes that are in complete LD with the 660K SNPs. The analysis of extremely large data set from a granddaughter design in dairy cattle shows clearly that with statistical evaluation of SNP chip and WGS information only, the identification of the causative quantitative trait nucleotide just based on concordance is not achieved (Weller et al., 2018). We therefore analyzed separately potentially functional SNPs in coding regions. In any case, because of high LD with the functional *VRTN* variants and extensive LD especially in L, it is impossible to disentangle the effect from these different alleles. Additional laboratory functional experiments would be needed.

### Phenotypic Differences Between Breeds

The primary task of the method used to sample NVE and RIB (Gangsei and Kongsro, 2016) is 3D segmentation of bones. Thus, the counts (NVE and RIB) utilized in the present study are just favorable by-products. The rib counting is challenging due to so-called half-ribs (Fredeen and Newman, 1962), underdeveloped ribs that are barely visible in the CT images. The rib count (RIB) might be viewed as a proxy variable for number of thoracic vertebrae. A higher proportion of underdeveloped ribs in L compared to D populations could be an additional explanation for the different effects observed for RIB between these two pig breeds.

**FIGURE 8:**
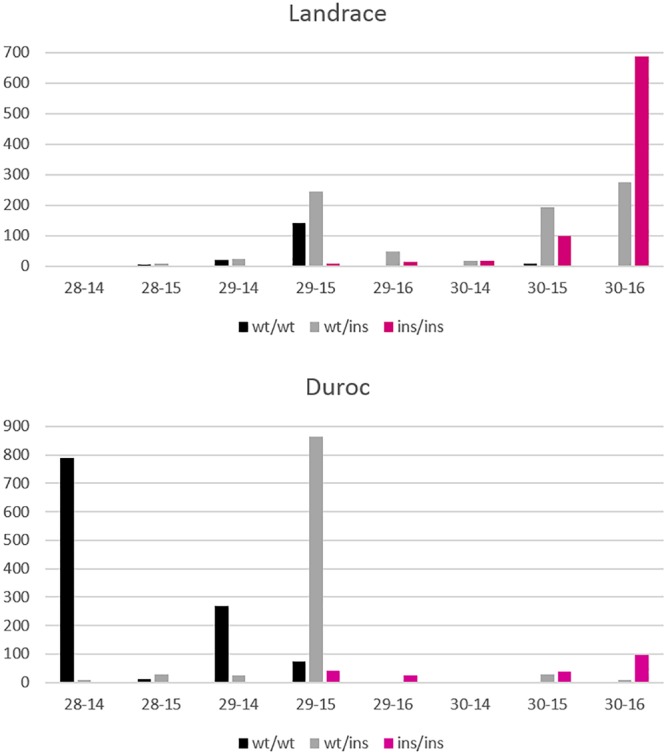
Distribution of NVE and RIB for Landrace and Duroc animals according to their genotypes wt/wt, wt/ins, and ins/ins at the VRTN gene. Number of animals is displayed on the y axis whereas number of vertebrae-ribs are on the x axis. A few animals with extreme phenotypes were discarded for better visibility.

Figure 8 shows the differences in allele frequencies at the *VRTN* locus in relation to the distribution of NVE and RIB for each breed. Homozygous wt/wt L animals have one NVE and RIB more than wt/wt D animals. In D, a large proportion of the animals are wt/ins and have 29 NVE with 15 RIB. The estimated effect comes mainly from this contrast between wt/wt and wt/ins animals. Very few animals are homozygous ins/ins and have 29 NVE with 15 RIB or 30 NVE with 16 RIB. However, nearly all L ins/ins animals show 30 NVE and the estimate of the allelic effect comes from the contrast between wt/ins and ins/ins animals. More than 60% of heterozygous L animals appear to already have 30 NVE and they show more variation in RIB. This indicates that in L animals other genetic factors are present causing a higher background level of NVE and RIB and causing more variability in RIB independent of *VRTN* genotype. We cannot disentangle whether additional mutations in LD with the long haplotype in the L breed cause the reduced effect on rib formation or whether other variants in the L breed blur the effect.

### Molecular Background of Life Development

In mammals, mammary gland complexes develop along a mammary line on each flank along the spine. The mammary line extends from the axilla to the inguin (Veltmaat, 2017). At designated points of the mammary line, mammary glands will develop in pairs. These points are determined by the underlying development of a vertebra. Vertebrae develop from the somites and mammary gland formation is initiated by factors in the dermal mesenchyme, which is also derived from the somites (Veltmaat et al., 2006). Further, each mammary gland is thought to be determined by specific genetic components, which determine whether its development will be initiated and continued.

**FIGURE 9:**
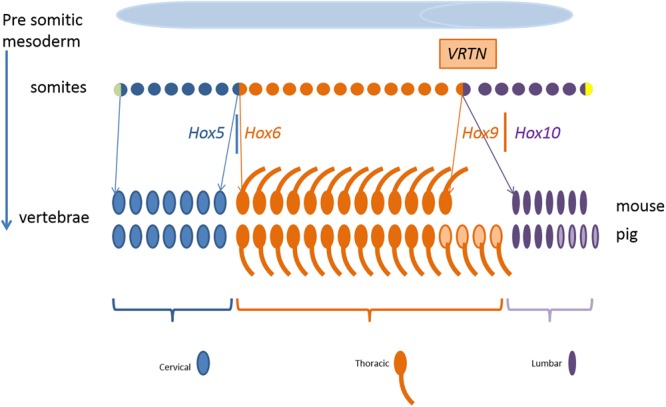
Development of the mouse and pig vertebral pattern from somites. Genes involved in the regulation and their spatial expression in italics. Variation in number of thoracic and lumbar vertebrae in pigs is indicated in light color. Parts of the figure was adopted from Gilbert (2000).

Figure 9 shows schematically the somites as progenitor cells of vertebrae, ribs, and mammary glands. Segmental identity of each somite is maintained by the Hox code which controls the positional specification of each segment that later forms, e.g., thoracic or lumbar vertebra (Wellik, 2007; Myers, 2008). In other words, the expression of Hox genes gradually changes along the axis from head to tail. For example, members of the paralog group Hox10 block rib formation whereas Hox6 proteins show rib promoting activity (Guerreiro et al., 2013).

In our QTL region on SSC7, the direct effect of the two *VRTN* variants initiate the development of an additional vertebra in ins/ins animals. VRTN has been identified as a DNA transcription factor increasing the expression of NOTCH2 and HES1 (hes family bHLH transcription factor 1) and is therefore involved in the regulation of the synchronized oscillation of the segmentation clock (Duan et al., 2018). This causes a change in the embryonic development rate increasing the number of somites but we observe also breed differences in the segmental identity. At the experimental level, increasing the number of cervical and thoracic vertebrae has been reported in transgenic mice by accelerating mRNA expression through reduction of number of introns of *HES7* (hes family bHLH transcription factor 7) gene (Harima et al., 2013). Their results also indicate that the link between the segmentation clock and the hox gene activation can be dissociated which causes a partial transformation of lumbar vertebrae into a thoracic vertebrae. They observe a shift in the anterior border of *HOXB6* (homeobox B6) and *HOXB9* (homeobox B9) expression by one or two somites in mice mutants with an accelerated expression of *HES7*. A partial transformation of lumbar vertebrae into a thoracic vertebra was also observed by knockout of *HOXC8* (homeobox C8) gene in mice (Le Mouellic et al., 1992). Even environmental influences such as reduced temperature during embryonic development have been reported to affect the segmentation clock in zebrafish under experimental conditions (Jiang et al., 2000). So both number of somites and type of vertebrae can be affected by gene variants in the NOTCH pathway. Depending on the genetic capacity of the individual at the other loci that regulate rib development the animal will also develop additional ribs.

Other QTL for NTE are detected in the GWAS that are most likely due to variation further downstream in the developmental cascade for the formation of the mammary gland. Different genetic factors have been described regulating pairs of mammary glands at different locations and even between the left and right counterpart (Veltmaat et al., 2006; Rohrer and Nonneman, 2017). All pairs of mammary glands in mice have been shown to be different in terms of their timing of appearance, their molecular requirements, and their morphogenetic program (Veltmaat, 2017).

### Identification of Other Functional Mutations

Analysis of the sequence variation in the SSC7 QTL region suggested that protein coding variants are unlikely to be having a large impact on the observed phenotypes. However, we identified two missense mutations in the *ABCD4* gene located just upstream of *VRTN*. One missense variant has a SIFT code of 0.03 and is therefore expected to be deleterious for function of the protein. These two variants together with a large list of non-coding variants are present in all L wt/wt animals. Obviously, these mutations accumulated in the L breed and are not present in the D breed. These mutations could be altering *ABCD4*, possibly changing its expression or function. Intriguingly there is seemingly a link between ABCD4 and the development of the spine. ABCD4 is believed to play a role in the intracellular processing of vitamin B12, and mutations affecting the ATPase domain of this protein have been shown to alter intracellular vitamin B_12_ trafficking (Coelho et al., 2012; Fettelschoss et al., 2017). Vitamin B_12_ is required as a cofactor in methionine synthase, and low levels of vitamin B_12_ during development are associated with higher levels of neural tube defects in humans (Ray and Blom, 2003; Groenen et al., 2004; Turner, 2018). With the addition of expression data, in future analyses it may be possible to better characterize variants in this region and identify other important functional mutations. The obvious sequence differences between the two breeds on the wildtype haplotypes could also explain why the size of the effect on NVE is larger in D than in the L breed.

### Origin of *VRTN* Promoter SNP and Insertion

The insertion allele characterized by the PRE1 insertion element and the SNP in the promoter of *VRTN* are only 1.2 kb apart. Fan et al. (2013) describe both *VRTN* variants to be in complete LD in three experimental populations of Western and Chinese origin. The insertion allele segregates in some Chinese breeds but Chinese wild boar and most Chinese indigenous breeds are wt/wt. Also in our data set, both *VRTN* mutations are in strong LD, but Zhang et al. (2016) describe an experimental cross where only the promoter SNP is segregating. To the contrary, among our sequenced animals, we do not find the promoter SNP variant without the insertion. However, we do find the insertion without the promoter SNP in LW, showing that recombinant animals are segregating in the LW breed. These recombinant animals (1.8% frequency) could be used to estimate the effect of the insertion separately in the future to test whether the increase in *VRTN* expression caused by the insertion alone is sufficient to generate an additional vertebra. Zhang et al. (2016) do not report an estimate of the allelic effect for the promoter SNP and describe that the effect in their data set is only due to a mutation in the *LTBP2* gene further distal on SSC7.

## Conclusion

In this study, a clear relationship between formation of the vertebral column and development of teats is observed in two populations of commercial pigs, L and D, differing largely in NTE. In both breeds, this difference in NTE is partly due to genetic variation in a region on SSC7, which has earlier been reported in several studies. By refining phenotype and examining NVE, noise from other loci is omitted, increasing overall heritability and significance of the SSC7 QTL region. However, the effect of two previously reported *VRTN* variants on thoracic vertebrae was found to be dependent on the genetic background. Allele frequencies at the QTL, size of the effect and accuracy of the phenotype differ between breeds and thereby influence genetic and phenotypic variance. Also, the overall population mean for RIB differs between L and D breeds. At the molecular lever, the large number of non-coding and coding variants observed to be present only in L on the wildtype haplotype show that the genetic background in the 3 Mb region encompassing *VRTN* differs between the two breeds. Moreover, other more subtle differences, such as variants affecting *ABCD4*, can also be expected in the remainder of the genome although they were not detectable in the GWAS results. The relationship between quantitative genetic parameters and the underlying factors of the developmental cascade of skeletal and mammary gland development gives a good example how biological factors influence our population parameters used for practical breeding value estimation. Identification and proof of causative mutations for oligogenic or polygenic traits without functional laboratory studies remains nearly impossible, especially for non-coding variants.

## Data Availability

The datasets analyzed for this study are included in the manuscript and/or the Supplementary Material.

## Ethics Statement

The data used for this study were obtained as part of routine data recording in a commercial breeding program. Samples collected for DNA extraction were only used for routine diagnostic purposes of the breeding program. Data recording and sample collection were conducted strictly in line with the rules given by Dutch and Norwegian Animal Research Authorities.

## Author Contributions

MS performed SNP detection in WGS data, was involved in imputation and haplotype work, and contributed to writing the paper. ML calculated genetic parameters, performed GWAS analyses, and was involved in writing the paper. HM performed functional SNP analyses, performed analyses of the LTBP2, and contributed to writing the paper. MD performed haplotype analyses and contributed to writing the paper. LG and JK performed phenotyping using CT images and was involved in writing the paper. MW and EG was involved in planning the project and contributed to writing the paper. BH was involved in planning the project, coordinated the studies, and drafted the paper. All authors have read and approved the final manuscript.

## Conflict of Interest Statement

MS, JK, and EG were employed by the company Norsvin SA. ML and BH were employed by the company Topigs Norsvin. LG was employed by the company Animalia AS. The remaining authors declare that the research was conducted in the absence of any commercial or financial relationships that could be construed as a potential conflict of interest.

## Abbreviations

ABCD4: ATP binding cassette subfamily D member 4 gene
CT: Computer tomography
D: Duroc
HES1: Hes family bHLH transcription factor 1 gene
HES7: Hes family bHLH transcription factor 7 gene
HOXB6: Homeobox B6 gene
HOXB9: Homeobox B9 gene
HOXC8: Homeobox C8 gene
IGV: Integrative Genomics Viewer software
L: Landrace
LD: Linkage disequilibrium
LTBP2: Latent transforming growth factor binding protein 2 gene
LW: Large White
NTE: Number of teats
NVE: Number of vertebrae
RIB: Number of ribs
SSC7: Sus scrofa chromosome 7
VRTN: Vertnin gene
VSX2: Visual system homeobox 2
WGS: Whole genome sequence
