# Supplementary material for: A QTL for Number of Teats Shows Breed Specific Effects on Number of Vertebrae in Pigs: Bridging the Gap Between Molecular and Quantitative Genetics
Source: Front Genet. 2019 Mar 26;10:272. doi: 10.3389/fgene.2019.00272 (PMC6445065; doi:10.3389/fgene.2019.00272)
Supplement: Supplementary file 6 [file Table_6.docx]

***Supplementary material***

**A QTL for number of teats shows breed specific effects on number of vertebrae in pigs: Bridging the gap between molecular and quantitative genetics**

**Maren van Son^1*^, Marcos S Lopes^2,3^, Henry J Martell^4^, Martijn F L Derks^5^, Lars Erik Gangsei^6,7^, Jorgen Kongsro^1^, Mark N Wass^4^, Eli H Grindflek^1^, Barbara Harlizius^2^**

***Correspondence:**

Corresponding Author

# [maren.van.son@norsvin.no](mailto:maren.van.son@norsvin.no)

**1 Supplementary Figures**


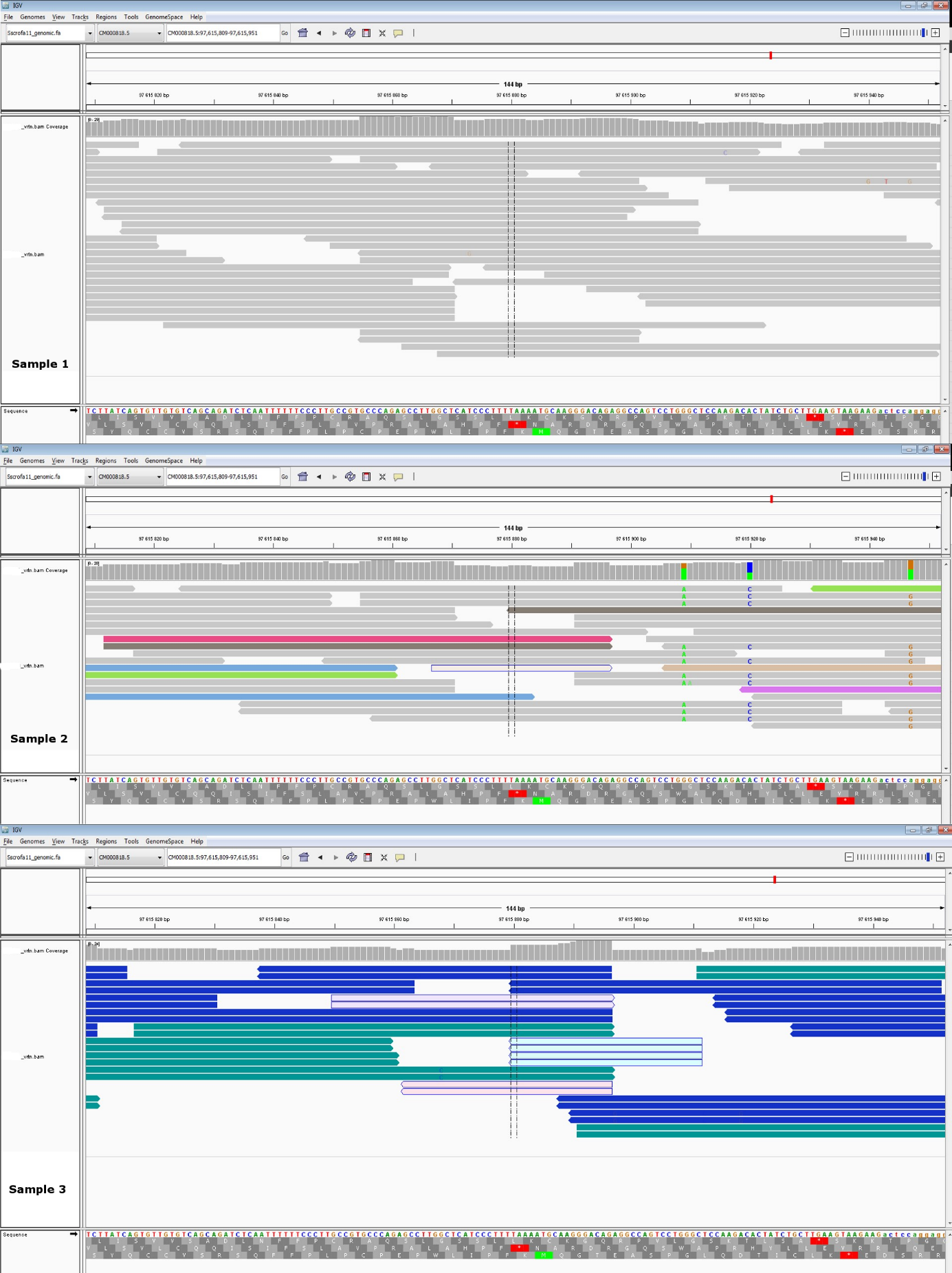


**Supplementary Figure S1. Genotyping of the *VRTN* insertion using IGV.** Three animals are shown in this picture: one wt/wt, one wt/ins and one ins/ins (*g.20311_20312ins291*). The grey reads are perfectly matching the reference sequence, indicating wild type allele, whereas the colored reads have one of the pairs in the paired end sequence mapping in this region and the other pair on a different chromosome, caused by the occurence of PRE1 SINEs several times in the genome and showing the position of the insertion. Some reads have also been clipped and are shorter because only a part of them match the reference sequence due to the insertion. The 24 first bases of the insertion are the same as the reference sequence and it is therefore an overlap of reads that start and end at the duplication breakpoint.


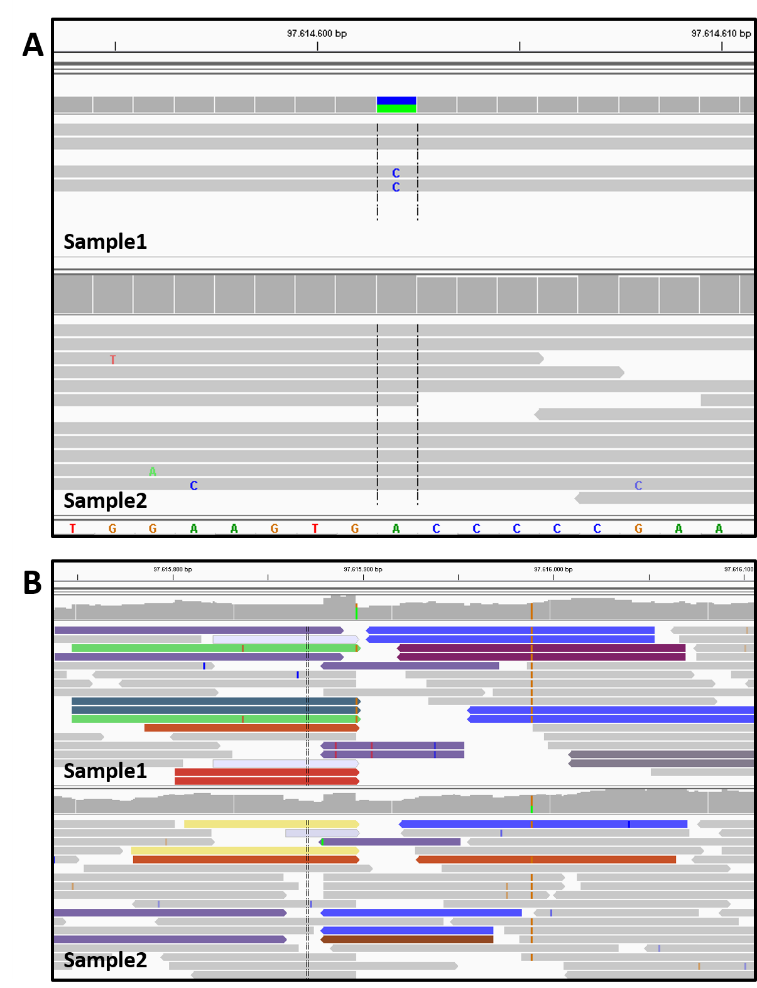


Supplementary Figure S2. IGV screen capture of the alignments for two recombinant sequenced samples in LW showing the genotypes of: A) the *g.19034A>C* *VRTN* promoter mutation, B) the VRTN *g.20311_20312ins291* PRE1-SINE element. Sample1 is heterozygous (AC) for the promotor SNP, but homozygous for the PRE1 insertion (all reads are clipped, no read-throughs), while Sample2 is wild-type AA for the promotor SNP, and heterozygous for the PRE1 insertion (supported by complete read-throughs). Color indicates reads for which the mate maps to a different chromosome, often the case for small SINE element insertions, which are spread throughout the genome.

**2 Supplementary Tables**

**Supplementary File S1. GWAS results from the 660K analysis.** Significant SNPs and QTL regions (*p*-values < 10^-06^) for NTE (Table X1), NVE (Table X2) and RIB (Table X3). The SNPs are described with chromosomal position, alleles (A1 and A2), frequency of A1, effect of A1 (b), standard error of b, *p*-value (p), log10 *p*-value, explained genetic variance (expl_VG), explained phenotypic variance (expl_VP), breed, trait, chip and QTL region.

**Supplementary File S2. GWAS results from the WGS fine mapping analysis of SSC7.** Significant SNPs with LD > 0.8 with the top SNP for NVE (Table X1) and RIB (Table X2). The SNPs are described with chromosomal position, alleles (A1 and A2), frequency of A1, effect of A1 (b), standard error of b, *p*-value (p), log10 *p*-value, LD to top SNP, explained genetic variance (expl_VG), explained phenotypic variance (expl_VP), breed, trait and chip (here: WGS data).

**Supplementary File S3. Human to pig *LTBP2* regulatory region mapping and WGS data for samples grouped by *VRTN* genotype.** Table X1) Mapping of known human *LTBP2* regulatory regions to the pig genome. Human coordinates are in reference to GRCh38, and pig coordinates are in reference to *Sus scrofa* build 11.1. Table X2) Gene focused summary of the WGS data, when grouping samples by genotype for the *VRTN* insertion (*g.20311_20312ins291*). This table summarizes the unique mutations in each gene, including the differences between genotype groups for shared variants, and the frequencies of variants unique to one genotype group. Variants are presented as the number of samples that have them, and then in brackets the mutation identifier and its consequence. Table X3) Summary of the variants found in the WGS data that are unique to the wt/wt genotype. Variants are ordered from most to least common. Table X4) Summary of the variants found in the WGS data that are unique to the wt/ins genotype. Variants are ordered from most to least common. Table X5) Summary of the variants found in the WGS data that are unique to the ins/ins genotype. Variants are ordered from most to least common. Table X6) Summary of the variants found in the WGS data in all three genotype groups. Variants are ordered from highest to lowest difference between the genotype groups. Table X7) Variants causing protein coding changes in the region, none of which are associated with any of the groups.

**Supplementary File S4. Haplotype analysis in Duroc.** Haplotypes were constructed in the SSC7 QTL region using medium density (Table X1-X2) and high density (Table X3-X4) SNP data. Table X1 shows the 80K SNPs in the QTL region and Table X2 shows the haplotypes constructed with these SNPs. Table X3 shows the 660K SNPs in the QTL region, as well as their *p*-values for NVE, and Table X4 shows the haplotypes constructed with these SNPs. For both 80K and 660K, the green haplotype is associated with the two functional *VRTN* mutations, and shows clear association with the number of vertebrae. Moreover, the six most significant SNPs from the GWAS are underlined. The two functional *VRTN* mutations are located in this core sequence underlined.

**Supplementary File S5. Landrace specific haplotype for the animals without the *VRTN* insertion.** The table shows SNPs included in a Landrace specific haplotype occurring in animals not having the *VRTN* insertion (wt/wt) (Table X1). Results from VEP are shown together with the genotypes of Landrace and Duroc animals sorted by their *VRTN* genotype. Table X2 shows the 660K chip SNPs in the region with alleles segregating with the *VRTN* wt vs. insertion genotype.
